# Morphologic analysis of the foveal avascular zone for prediction of postoperative visual acuity in advanced idiopathic epiretinal membrane

**DOI:** 10.1038/s41598-023-35948-1

**Published:** 2023-06-27

**Authors:** Gee-Hyun Kim, Bo-Een Hwang, Heejeong Chun, Joo Young Kim, Rae Young Kim, Mirinae Kim, Young-Geun Park, Young-Hoon Park

**Affiliations:** 1 Department of Ophthalmology and Visual Science, Seoul St. Mary’s Hospital, College of Medicine, The Catholic University of Korea, 222, Banpo-daero, Seocho-gu, Seoul, 06591 Republic of Korea; 2 Catholic Institute for Visual Science, College of Medicine, The Catholic University of Korea, Seoul, Republic of Korea

**Keywords:** Retinal diseases, Optical techniques, Prognostic markers

## Abstract

To investigate the preoperative morphology of the foveal avascular zone (FAZ) for prediction of the postoperative visual acuity in advanced idiopathic epiretinal membrane (ERM). 28 patients (28 eyes) with unilateral idiopathic ERM who underwent pars plana vitrectomy with internal limiting membrane peeling were included. Superficial FAZ was measured preoperatively in both eyes using optical coherence tomography angiography. Area, perimeter, and circularity of FAZ were achieved, and the differences between the ERM eyes and the contralateral eyes were evaluated to analyze the degree of FAZ distortion in diseased eyes. The best-corrected visual acuity (BCVA) and central foveal thickness (CFT) were measured at baseline and more than 6 months after surgery. The correlations of the preoperative FAZ with BCVA and CFT were assessed. The FAZ in the eyes with ERM was significantly reduced, and the BCVA was significantly correlated with the FAZ area (FAZa) (*P* = 0.001) and the FAZ perimeter (FAZp) (*P* < 0.001) before surgery. LogMAR BCVA and CFT were significantly improved from 0.550 ± 0.221 to 0.354 ± 0.229 (*P* = 0.008), and from 524.393 ± 93.575 µm to 400.071 ± 75.979 µm (*P* < 0.001) after surgery. The preoperative FAZa and FAZp were significantly associated with letter score gain (*P* < 0.001, *P* < 0.001) and the postoperative final BCVA (*P* = 0.026, *P* = 0.006). The preoperative FAZp had correlation with ratio of postoperative to preoperative CFT (*P* = 0.016). The preoperative FAZp is a predictor of visual acuity and morphological prognosis after surgery in advanced idiopathic ERM.

## Introduction

Idiopathic epiretinal membrane (ERM) is a common retinal disease^1^. An ERM is fibro-cellular proliferation over the internal limiting membrane (ILM) causing morphological distortion of macula which may lead to metamorphopsia and/or deterioration of visual acuity^1^. The incidence of ERM among Asians was reported as 12.1%, and the rate depends on age and axial length^2^.

Pars plana vitrectomy with ERM removal is the mainstay of treatment to reduce traction on the retina for improving morphology and function. The ILM is usually peeled together to avoid the recurrence of ERM^3^. Despite the anatomical improvement after surgery, patients may have persistent visual disturbance. Therefore, several preoperative factors have been researched to predict postoperative visual outcome. Lately, preoperative inner retinal deformation was known to be correlated with postoperative visual improvement^4^.

Since the early 1990s, optical coherence tomography (OCT) has evolved as an important diagnostic modality for macular diseases. It helps precise and extensive observation of the retinal structures, so the correlation of visual function with distortion of the outer retinal layer could be revealed. In ERM, structural changes of interdigitation and ellipsoid zone within the foveola were first suggested as prognostic factors^5–9^. Moreover, OCT has been used to elucidate the correlation of metamorphopsia with structural changes such as thickening of an ectopic inner retinal layer in the fovea due to ERM traction^10–14^, and inner retinal changes affecting the pathway of blood vessels^11–15^. Although the relationship between the ERM-associated visual disturbance and the retinal structural changes has been revealed by OCT, there is lack of consensus about optimal timing of ERM surgery^13^.

Currently, OCT angiography (OCTA) has been renowned as a minimally invasive method for evaluating retinal vessels. Its tomographic nature allows visualization of vascular plexuses layer by layer. It is useful not only in diagnosis of retinal blood vessel diseases, but also in measuring of the foveal avascular zone (FAZ)^16,17^. Increase in foveal and decrease in parafoveal vascular density have been discovered by OCTA in eyes with ERM before surgery and vice versa after surgery^18,19^. Some recent studies using OCTA have reported the FAZ enlarges and becomes more circular after surgery in eyes with ERM^19–22^. However, there is something unrevealed between FAZ morphology and postoperative visual function. Using OCTA in this study, we measured area, perimeter, and circularity of the FAZ before ERM surgery and investigated their associations with postoperative visual acuity.

## Methods

### Study population

This is a retrospective observational study which was conducted in the Department of Ophthalmology and Visual Science at Seoul St. Mary’s Hospital, the Catholic University of Korea. This study adheres to the tenets of the Declaration of Helsinki. All protocols were approved by the Institutional Review Board (IRB) of the Catholic University of Korea (KC23RASI0092). Written informed consent procedures were exempted based on the provisions of the IRB, due to the retrospective nature and data anonymization of the study.

28 patients with idiopathic ERM suffering from decrease of visual acuity generally with metamorphopsia detected by Amsler grid findings underwent PPV with ILM peeling and perfluoropropane (C_3_F_8_) gas tamponade at our clinic from 2021 September to 2022 July.

We excluded patients with a history of ocular trauma, retinal surgery, or intravitreal injection. Also, cases which had diabetic retinopathy, retinal vessel occlusion, ocular inflammatory disease, or any other vitreoretinal and anterior segment diseases were excluded for reliability of data. Those with prior optic neuropathy, glaucoma suspect, ocular hypertension, glaucoma, or significant media opacity were also excluded. We excluded patients with severely compromised visual acuity whose decimal BCVA was under 0.05.

### Study protocol

At the initial visit, all subjects’ demographic data, medical and ophthalmologic history were collected. They were examined by slit-lamp microscopy, and dilated fundus examination was also done. Axial length was measured using IOL-Master 700 (Carl Zeiss Meditec, Jena, Germany), and OCTA imaging was performed by DRI Triton SS-OCT (Topcon, Tokyo, Japan) before surgery. Snellen BCVA and OCT images were achieved, and presence or absence of patients' metamorphosia was confirmed by Amsler grid before the surgery. At postoperative follow-ups, BCVA was measured, and OCT was performed until 6 months from the surgery after the gas tamponade was absorbed.

All OCT and OCTA images were reviewed and analyzed independently, and each measurement was averaged from two experienced independent retinal specialists (Y-H.P. and G-H.K.) who were blinded to each other and to the clinical histories of subjects. Normal contralateral eyes of all subjects were also evaluated as controls considering that the morphology of the FAZ could be influenced by the patient’s demographic characteristics.

### Surgical technique

Under general anesthesia, a standard 25-gauge 3-port PPV (Constellation device, Alcon, Fort Worth, TX, USA) was conducted by the same experienced surgeon (Y-H.P.). Phacoemulsification and posterior chamber intraocular lens (Artis PL E; Cristalens Industrie, Lannion, France) implantation were performed before if necessary. In every patient, vitrectomy was fully performed, so that posterior hyaloid was elevated and trimmed until the peripheral vitreous base. A macular ERM was peeled, and ILM was also peeled off with forceps in an area of at least 2-disc diameter around the fovea after 0.05 mL of 0.05% indocyanine green (Diagno green; Cheil Pharm. Co., Korea) were applied on the macula for one minute. The peripheral retina was thoroughly inspected, and barrier photocoagulation by argon laser was conducted around any degenerative lesions, retinal tears or holes. Finally, the vitreous cavity was filled with 10–14% C_3_F_8_ gas in every case. Patients were mandated to be in prone position at least for a week after the surgery.

### OCT/OCTA image analysis

Preoperative OCT/OCTA images were acquired for both eyes the day before the operation. Postoperative OCT images were taken for the ERM eyes after more than 6 months from the operation day. Image qualities of all OCT scans were above 65 and OCTA scans above 50. Images with motion artifacts and/or stretch artifacts were also excluded. Mean image quality of OCT was 89.07, and OCTA was 64.43 in this study.

**Figure 1:**
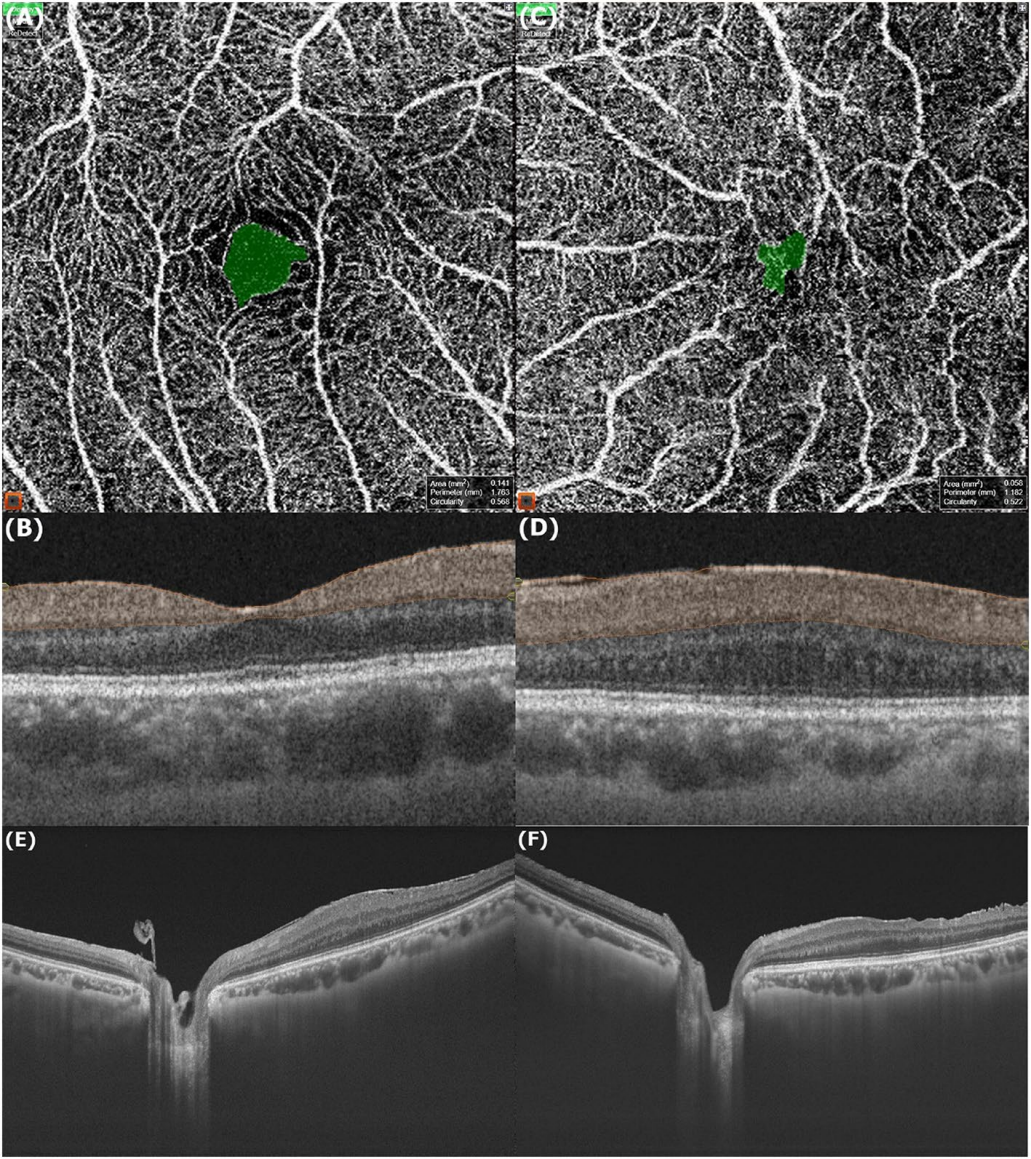
Representative images from OCTA and swept-source OCT. Images of the preoperative FAZ (green area on images) (**A**) and measurement range (orange zone, from internal limiting membrane to the inner plexiform layer) (**B**) of the control eye (area = 0.141 mm^2^, perimeter = 1.763 mm, circularity = 0.568); (**C**), (**D**) of the eye with ERM (area = 0.058 mm^2^, perimeter = 1.182 mm, circularity = 0.522); Preoperative macular morphology of the ERM eye (CFT = 552 µm) (**E**); Postoperative macular morphology (CFT = 315 µm) (**F**).

We analyzed the morphology of macular based on the 12 mm × 12 mm 3D scan mode SS-OCT images. ERM was staged by OCT-based 4-stage grading system which was proposed by Govetto et al.^23^ considering the importance of continuous ectopic inner foveal layers which is known to be associated with lower visual acuity. Pre- and postoperative CFT were measured manually using the digital caliper function featured in Topcon image-NET 6 software. Each measurement was averaged from two experienced independent retinal specialists. We obtained the preoperative FAZ for the superficial layers, between the ILM and the inner plexiform layer, by analyzing 4.5 × 4.5 mm OCTA images with built-in tool of Topcon image-NET 6 software. We investigated the correlations of the preoperative FAZ parameter with pre- and postoperative visual acuity, letter score improvement, and changes in CFT. Representative cases are illustrated in Fig. 1.

### Statistical analysis

Continuous variables were presented as the mean ± standard deviation (SD). Normal distributions of the data were examined before choosing the statistical analysis methods. Differences from gender, laterality or presence of metamorphopsia of the study eye on other variables were inspected using Mann–Whitney U test. The measurements of BCVA were converted into the logarithm of the minimum angle of resolution (LogMAR) to analyze changes of BCVA after the operation. Functional and anatomical improvements were analyzed by Wilcoxon signed-rank test. Correlation analysis was performed to figure out any inappropriate dependency among preoperative parameters. Finally, univariate linear regression analysis was performed to show detailed correlations between presumed prognostic factors and postoperative outcomes. Statistical analysis was performed using the Statistical Package for the Social Sciences for Windows (Version 26.0, SPSS Inc., Chicago, IL). *P*-values of < 0.05 were considered as statistically significant.

## Results

Data from 28 eyes of 28 patients (5 males and 23 females) with an age range from 54 to 76 years (mean 63.1 ± 6.9 years) were included in this study. Only one eye (3.571%) was pseudophakia. The others (96.429%) had a clear phakic lens and underwent phacovitrectomy. 24 eyes (85.714%) suffer from metamorphopsia, but presence or absence of metamorphosis was not associated with significant differences in other variables included in this study. Demographics, preoperative characteristics of the study eyes are provided in Table 1. Generally, the FAZ was significantly contracted (*P *< 0.001) and distorted (*P* = 0.003) compared with the contralateral eye preoperatively as shown in Table 2. The BCVA was significantly improved after surgery compared with the preoperative values (*P* = 0.008). CFT was selected as a representative parameter of the morphology of ERM, and there were significant differences between preoperative and postoperative measurements (*P* < 0.001) (Table 3). Intraclass correlation coefficient (ICC) for the manual measurements by two researchers (Y-H.P. and G-H.K.) was 0.985 (95% CI 0.968 ~ 0.993) for before operation, and 0.986 (95% CI 0.974 ~ 0.994) for after operation.

**Table 1:** Demographics of the patients and characteristics of the study eyes.

Total number of eyes	28
Age (years)	63.1 ± 6.9
Axial length (mm)	23.796 ± 1.336
Laterality (right : left)	11: 17
Sex (male : female)	5 : 23
Lens status (phakia : pseudophakia)	27 : 1
ERM stage (1/2/3/4)	0/2/7/19
Presence/absence of metamorphopsia	24/4

Data are presented as the mean ± standard deviation or a ratio, as appropriate.

ERM, Epiretinal membrane.

**Table 2:** Preoperative FAZ parameters in the ERM eyes.

	ERM eyes	Control eyes	Ratio	*P*
FAZa	0.144 ± 0.114	0.355 ± 0.081	0.415 ± 0.320	< 0.001
FAZp	1.714 ± 0.662	2.662 ± 0.366	0.653 ± 0.265	< 0.001
FAZc	0.540 ± 0.101	0.623 ± 0.060	0.879 ± 0.203	0.003

Data are presented as the mean ± standard deviation.

ERM, Epiretinal membrane; FAZ, Foveal avascular zone; FAZa, FAZ area; FAZp, FAZ perimeter; FAZc, FAZ circularity.

**Table 3:** Visual acuity and morphology before and after surgery in the ERM eyes.

	Preoperative	Postoperative	*P*
BCVA (LogMAR)	0.550 ± 0.221	0.354 ± 0.229	0.008
CFT (µm)	524.393 ± 93.575	400.071 ± 75.979	< 0.001

Data are presented as the mean ± standard deviation.

BCVA, Best-Corrected Visual Acuity; CFT, Central Foveal Thickness.

**Figure 2:**
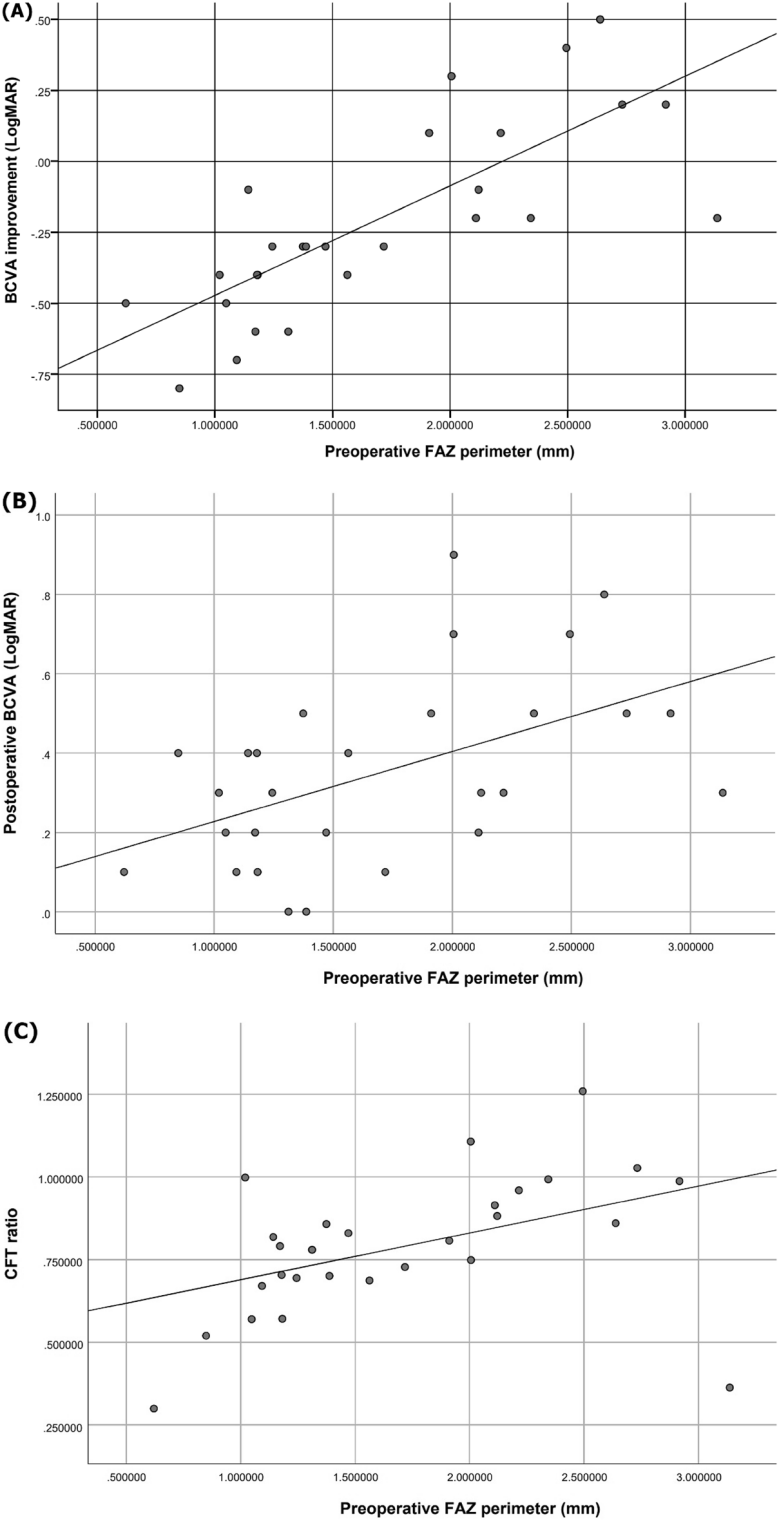
Scatterplots to exhibit correlations between preoperative FAZ perimeter versus BCVA improvement (LogMAR) (R^2^ = 0.574, *P* < 0.001) (**A**); postoperative BCVA (LogMAR) (R^2^ = 0.260, *P* = 0.006) (**B**); and CFT ratio (R^2^ = 0.203, *P* = 0.016) (**C**).

BCVA improvement was correlated with preoperative BCVA (r = − 0.739, *P* < 0.001), FAZa (r = 0.670, *P* < 0.001) and FAZp (r = 0.758, *P* < 0.001) (Table 4) (Fig. 2(A)). Preoperative BCVA was significantly correlated with preoperative FAZa (r = -0.587, *P* = 0.001) and FAZp (r = − 0.628, *P* < 0.001) (Table 4). Postoperative final BCVA was also significantly correlated with preoperative FAZa (r = 0.420, *P* = 0.026) and FAZp (r = 0.510, *P* = 0.006) (Table 4) (Fig. 2(B)).

**Table 4:** Correlations of visual acuity with preoperative parameters.

Preoperative	Preoperative BCVA (LogMAR)		Postoperative BCVA (LogMAR)		BCVA improvement (LogMAR)	
	r	*P*	r	*P*	r	*P*
BCVA (LogMAR)	–	–	− 0.123	0.532	− **0.739**	** < 0.001**
FAZa	− **0.587**	**0.001**	**0.420**	**0.026**	**0.670**	** < 0.001**
FAZp	− **0.628**	** < 0.001**	**0.510**	**0.006**	**0.758**	** < 0.001**
FAZc	− 0.141	0.474	− 0.299	0.122	− 0.110	0.576
Axial length	− 0.024	0.902	− 0.097	0.624	− 0.050	0.802

Statistically significant *P*-value is shown in bold.

BCVA, Best-Corrected Visual Acuity; FAZ, Foveal avascular zone; FAZa, FAZ area; FAZp, FAZ perimeter; FAZc, FAZ circularity; BCVA improvement (LogMAR) = Postoperative BCVA (LogMAR)—Preoperative BCVA (LogMAR).

r = correlation coefficient, *P* = significant value.

Preoperative CFT had significant correlations with preoperative BCVA (r = 0.670, *P* < 0.001), and FAZp (r = − 0.524, *P* = 0.004). We defined CFT ratio as a ratio of postoperative to preoperative CFT to avoid individual variations and to represent the morphological improvement after the surgical intervention. It was significantly correlated with preoperative BCVA (r = -0.437, *P* = 0.020), and FAZp (r = 0.450, *P* = 0.016) (Table 5) (Fig. 2(C)).

**Table 5:** Correlations of morphology with preoperative parameters.

Preoperative	Preoperative CFT		Postoperative CFT		CFT ratio	
	r	*P*	r	*P*	r	*P*
BCVA (LogMAR)	**0.670**	** < 0.001**	− 0.037	0.853	− **0.437**	**0.020**
FAZa	− 0.374	0.050	0.051	0.796	0.264	0.175
FAZp	− **0.524**	**0.004**	0.170	0.386	**0.450**	**0.016**
FAZc	0.099	0.617	− 0.299	0.122	− 0.326	0.090
Axial length	− 0.027	0.891	− 0.131	0.505	− 0.036	0.856

Statistically significant *P*-value is shown in bold.

BCVA, Best-Corrected Visual Acuity; FAZ, Foveal avascular zone; FAZa, FAZ area; FAZp, FAZ perimeter; FAZc, FAZ circularity; CFT, Central Foveal Thickness; CFT ratio = Postoperative CFT/Preoperative CFT.

r = correlation coefficient, *P* = significant value.

## Discussion

The indications for the surgical intervention for idiopathic ERM have not been agreed^24^. Surgical patient selection depends on the symptoms of patients, their visual requirements, and technical proficiency of the surgeon. But ERM patients usually have moderately good preoperative vision^24^. Therefore, the selection should be careful, and importance of preoperative predictors for postoperative visual outcome has been raised.

We obtained automated quantitative FAZ measurements on superficial plexuses by OCTA images with built-in tool of Topcon image-NET 6 software. In this study, the preoperative FAZ area was generally smaller and more distorted in the ERM eyes compared with the corresponding control eyes as reported previously. Preoperative FAZ reduction may reflect degree of retinal structural abnormality due to ERM contraction nearby the foveal center. In accordance with previous studies, we also found that eyes with thicker CFT prone to have smaller FAZ^22,25^. Yoshida et al.^1^ reported that there was inverse correlation between the FAZ area and stage of ERM, suggesting that the retinal structure changes both vertically and horizontally due to the contraction force by an ERM. And this is not just about the morphology but also about the visual function. In eyes with smaller FAZ, visual acuity was more severely compromised than eyes with relatively larger FAZ^25^. More contracted FAZ (smaller FAZa and FAZp) seems to be related with higher mechanical stress on the outer retina. A higher preoperative FAZ contraction rate was also associated with a more significant improvement in visual acuity after surger^1,25^. According to the previous study, FAZ was known to enlarge and become more circular postoperatively^1,25^. Hence, if the ERM is only associated with FAZ contraction but has not yet caused fragmentation of ellipsoid zone, surgical removal of the ERM may improve the visual acuity proportionally to the degree of the mechanical stress caused by the ERM. However, there is only one previous study about the relationship between preoperative FAZ measurements and final visual acuity after ERM surgery, and the result was also inconsistent because the correlation between preoperative FAZc and postoperative BCVA could be established only at 3 months after the operation^26^. According to the study, postoperative BCVA showed a trend of improvement even at 6 months after the operation^25^. Therefore, we measured the final postoperative BCVA at least after 6 months from the operation day in this study.

In this study, we unprecedentedly discovered the preoperative parameters (FAZa and FAZp) which show statistically significant correlation with the relative long-term final postoperative BCVA. In particular, FAZp showed very low *P*-values with every functional and morphological results. The reason for our new discovery would be the characteristics of our study population. Compared to the previous studies, which had mean baseline Snellen BCVA 0.48, 0.59, 0.63, and 0.34^1,22,24,26^, our study population has considerably lower preoperative visual acuity (Snellen BCVA 0.28). The improvement in visual acuity after surgery was statistically significant in this study, but the mean postoperative visual acuity (Snellen BCVA 0.44) was still lower than previous studies (Snellen BCVA 0.83, 0.74, 1.0, and 0.78)^1,22,24,26^. Our study population has generally more advanced ERM (stage 2 = 7.1%; stage 3 = 25.0%; stage 4 = 67.9%) and surgical patient selection in our clinic seems to be more conservative. Same as in previous studies, FAZa/p still significantly correlate with preoperative visual acuity and decorrelate with degree of improvement in visual acuity after surgery even in our study group of advanced ERM. Moreover, the absolute value of correlation coefficient is quite greater in the latter decorrelation between FAZa/p and improvement in visual acuity after surgery. As a result, if FAZa/p is huge, the final postoperative visual acuity becomes statistically worse in our study group. This whole relationship seems statistically more robust in case of FAZp.

**Figure 3:**
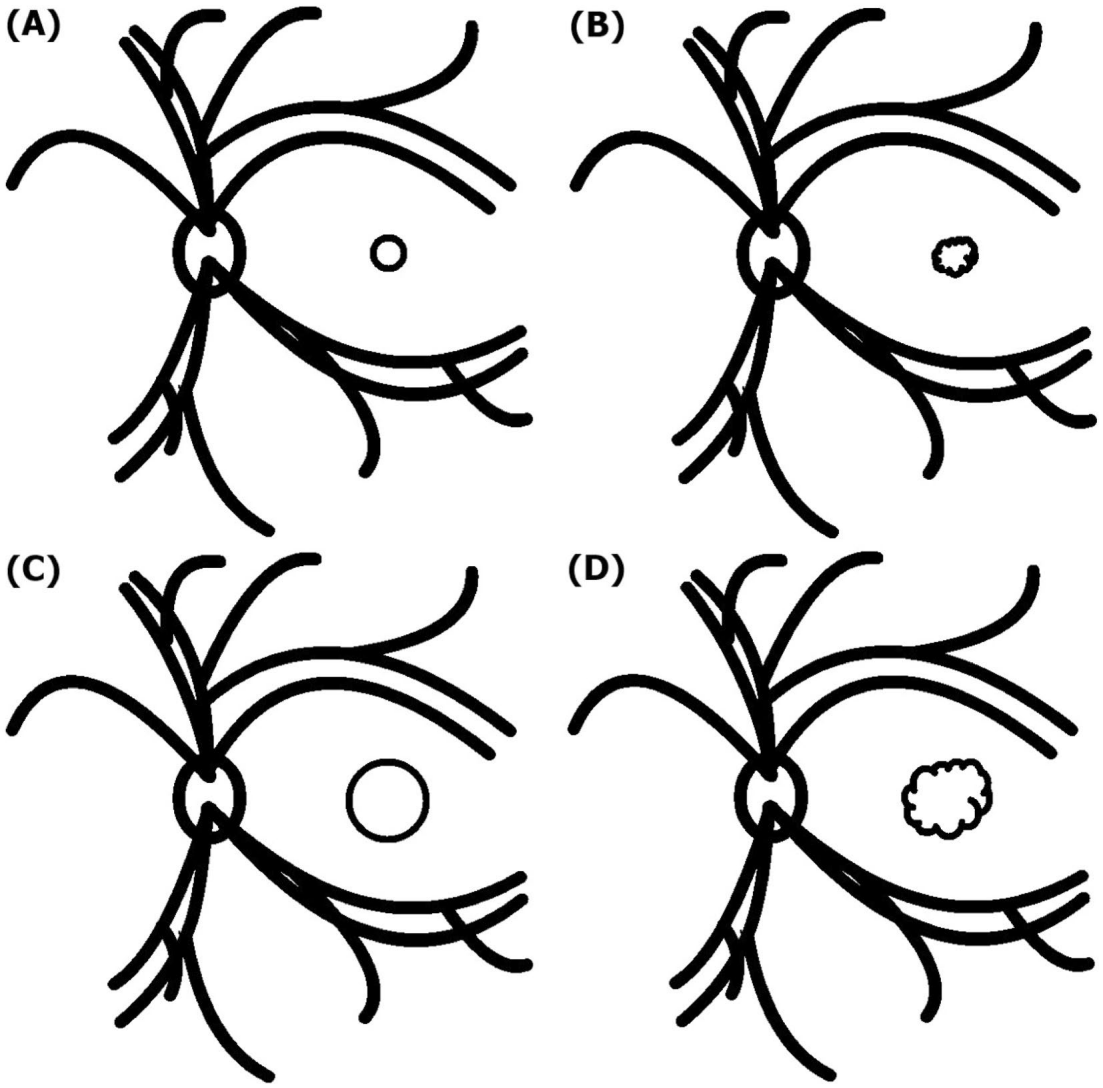
Four morphological categories of FAZ: small and circular (**A**); small and irregular (**B**); large and circular (**C**); large and irregular (**D**).

Geometrically, shape of FAZ can be categorized into four types; small and circular; small and irregular; large and circular; large and irregular (Fig. 3). In the population of more severe ERM patients, proportion of large but circular (normal) FAZ would decrease. Then, long perimeter of FAZ statistically means large and irregular FAZ in this population. Therefore, strong decorrelation between preoperative FAZp and postoperative visual acuity can be established.

Our new discovery would give theoretical understanding about statistics of FAZ morphology in ERM and provide practical application to the clinics of conservative surgical patient selection. Especially, technical simplicity of our pragmatic method would facilitate actual use in the clinical office.

There are some limitations in this study. First one is its retrospective nature with a relatively small number of cases and a short follow-up period. Second, errors in segmentation process may occur due to disruption of the retinal structure by ERM contraction, and this leads to incorrect FAZ analysis. Using built-in automatic tool of Topcon image-NET 6 software, our FAZ analysis could be free from human bias, but, on the other hand, it might suffer from inadvertent technical errors. In the future, more cases should be analyzed including not only the superficial retinal plexuses but also the deep retinal vessels. Third, postoperative FAZ analysis was not conducted in our study for economic reasons. Despite consistent results from previous studies about comparison between pre- and postoperative FAZ in ERM^1,25^, it would be better to verify it again in our study. Fourth, we only checked presence or absence of patients’ metamorphopsia before the surgery. If there was more quantitative evaluation of horizontal and vertical metamorphopsia such as M-CHARTS (Inami, Tokyo, Japan), we could analyze various aspects of visual function in ERM patients. However, according to the previous study^1^, there was no correlation between the FAZ area and postoperative M-CHARTS score which is concordance with our limited analysis to some extent. Further evaluation of various stages of ERM cases using OCTA and M-CHARTS before and after surgery would provide deeper understanding of our new findings.

## Data Availability

The datasets used and/or analyzed during the current study are available from the corresponding author on reasonable request.

## References

[1] Yoshida H (2020). Relationship between morphological changes in the foveal avascular zone of the epiretinal membrane and postoperative visual function. BMJ Open Ophthalmol..

[2] Cheung N (2017). Prevalence and risk factors for epiretinal membrane: The Singapore Epidemiology of Eye Disease study. Br. J. Ophthalmol..

[3] Azuma K, Ueta T, Eguchi S, Aihara M (2017). Effects of internal limiting membrane peeling combined with removal of idiopathic epiretinal membrane: A systematic review of literature and meta-analysis. Retina.

[4] Jeon S, Jung B, Lee WK (2019). Long-term prognostic factors for visual improvement after epiretinal membrane removal. Retina.

[5] Kim JH, Kim YM, Chung EJ, Lee SY, Koh HJ (2012). Structural and functional predictors of visual outcome of epiretinal membrane surgery. Am. J. Ophthalmol..

[6] Shimozono M (2012). The significance of cone outer segment tips as a prognostic factor in epiretinal membrane surgery. Am. J. Ophthalmol..

[7] Inoue M (2010). Inner segment/outer segment junction assessed by spectral-domain optical coherence tomography in patients with idiopathic epiretinal membrane. Am. J. Ophthalmol..

[8] Itoh Y, Inoue M, Rii T, Hirota K, Hirakata A (2013). Correlation between foveal cone outer segment tips line and visual recovery after epiretinal membrane surgery. Invest. Ophthalmol. Vis. Sci..

[9] Watanabe K, Tsunoda K, Mizuno Y, Akiyama K, Noda T (2013). Outer retinal morphology and visual function in patients with idiopathic epiretinal membrane. JAMA Ophthalmol..

[10] Okamoto F, Sugiura Y, Okamoto Y, Hiraoka T, Oshika T (2015). Inner nuclear layer thickness as a prognostic factor for metamorphopsia after epiretinal membrane surgery. Retina.

[11] Koo HC, Rhim WI, Lee EK (2012). Morphologic and functional association of retinal layers beneath the epiretinal membrane with spectral-domain optical coherence tomography in eyes without photoreceptor abnormality. Graefes Arch. Clin. Exp. Ophthalmol..

[12] Ichikawa Y, Imamura Y, Ishida M (2018). Inner nuclear layer thickness, a biomarker of metamorphopsia in epiretinal membrane, correlates with tangential retinal displacement. Am. J. Ophthalmol..

[13] Govetto A, Lalane RA, Sarraf D, Figueroa MS, Hubschman JP (2017). Insights into epiretinal membranes: Presence of ectopic inner foveal layers and a new optical coherence tomography staging scheme. Am. J. Ophthalmol..

[14] Watanabe A, Arimoto S, Nishi O (2009). Correlation between metamorphopsia and epiretinal membrane optical coherence tomography findings. Ophthalmology.

[15] Okamoto F, Sugiura Y, Okamoto Y, Hiraoka T, Oshika T (2012). Associations between metamorphopsia and foveal microstructure in patients with epiretinal membrane. Invest. Ophthalmol. Vis. Sci..

[16] Cicinelli MV (2017). Clinical spectrum of macular-foveal capillaries evaluated with optical coherence tomography angiography. Retina.

[17] Freiberg FJ (2016). Optical coherence tomography angiography of the foveal avascular zone in diabetic retinopathy. Graefes Arch. Clin. Exp. Ophthalmol..

[18] Nelis P, Alten F, Clemens CR, Heiduschka P, Eter N (2017). Quantification of changes in foveal capillary architecture caused by idiopathic epiretinal membrane using OCT angiography. Graefes Arch. Clin. Exp. Ophthalmol..

[19] Chen H (2019). Macular microvasculature features before and after vitrectomy in idiopathic macular epiretinal membrane: An OCT angiography analysis. Eye (Lond.).

[20] Kitagawa Y, Shimada H, Shinojima A, Nakashizuka H (2019). Foveal avascular zone area analysis using optical coherence tomography angiography before and after idiopathic epiretinal membrane surgery. Retina.

[21] Hirata A (2019). Relationship between the morphology of the foveal avascular zone and the degree of aniseikonia before and after vitrectomy in patients with unilateral epiretinal membrane. Graefes Arch. Clin. Exp. Ophthalmol..

[22] Kim YJ, Kim S, Lee JY, Kim JG, Yoon YH (2018). Macular capillary plexuses after epiretinal membrane surgery: An optical coherence tomography angiography study. Br. J. Ophthalmol..

[23] Govetto A, Lalane RA, Sarraf D, Figueroa MS, Hubschman JP (2017). Insights into epiretinal membranes: Presence of ectopic inner foveal layers and a new optical coherence tomography staging scheme. Am J Ophthalmol..

[24] Kunikata H, Abe T, Kinukawa J, Nishida K (2011). Preoperative factors predictive of postoperative decimal visual acuity ≥ 1.0 following surgical treatment for idiopathic epiretinal membrane. Clin. Ophthalmol..

[25] Bringmann A, Wiedemann P (2009). Involvement of Müller glial cells in epiretinal membrane formation. Graefes Arch. Clin. Exp. Ophthalmol..

[26] Bacherini D (2021). The role of OCT angiography in the assessment of epiretinal macular membrane. J. Ophthalmol..

